# Effects of vitamin D supplementation on FGF23: a randomized-controlled trial

**DOI:** 10.1007/s00394-018-1672-7

**Published:** 2018-03-30

**Authors:** Christian Trummer, Verena Schwetz, Marlene Pandis, Martin R. Grübler, Nicolas Verheyen, Martin Gaksch, Armin Zittermann, Winfried März, Felix Aberer, Julia Steinkellner, Claudia Friedl, Vincent Brandenburg, Jakob Voelkl, Ioana Alesutan, Barbara Obermayer-Pietsch, Thomas R. Pieber, Andreas Tomaschitz, Stefan Pilz

**Affiliations:** 1 Division of Endocrinology and Diabetology, Department of Internal Medicine, Medical University of Graz, Auenbruggerplatz 15, 8036 Graz, Austria; 2 Department of Cardiology, Swiss Cardiovascular Center Bern, Bern University Hospital, University of Bern, Freiburgstraße 4, 3010 Bern, Switzerland; 3 Division of Cardiology, Department of Internal Medicine, Medical University of Graz, Auenbruggerplatz 15, 8036 Graz, Austria; 4 Department of Laboratory Medicine, Paracelsus Medical University, Müllner Hauptstraße 48, 5020 Salzburg, Austria; 5 Clinic for Thoracic and Cardiovascular Surgery, Heart and Diabetes Center NRW, Georgstraße 11, 32545 Bad Oeynhausen, Germany; 6 Clinical Institute of Medical and Chemical Laboratory Diagnostics, Medical University of Graz, Auenbruggerplatz 15, 8036 Graz, Austria; 7 Division of Nephrology, Department of Internal Medicine, Medical University of Graz, Auenbruggerplatz 27, 8036 Graz, Austria; 8 Department of Cardiology, University Hospital of the RWTH Aachen, Pauwelsstraße 30, 52074 Aachen, Germany; 9 Medizinische Klinik mit Schwerpunkt Kardiologie, Campus Virchow-Klinikum, Charité-Universitätsmedizin Berlin, Augustenburger Platz 1, 13353 Berlin, Germany; 10 CBmed, Center for Biomarker Research in Medicine, Stiftingtalstraße 5, 8010 Graz, Austria; 11 Bad Gleichenberg Clinic, Schweizerei Weg 4, 8344 Bad Gleichenberg, Austria

**Keywords:** Vitamin D supplementation, FGF23, RCT, Phosphate, Calcitriol

## Abstract

**Purpose:**

Fibroblast growth factor-23 (FGF23) is critical for phosphate homeostasis. Considering the high prevalence of vitamin D deficiency and the association of FGF23 with adverse outcomes, we investigated effects of vitamin D3 supplementation on FGF23 concentrations.

**Methods:**

This is a post-hoc analysis of the Styrian Vitamin D Hypertension trial, a single-center, double-blind, randomized, placebo-controlled trial, conducted from 2011 to 2014 at the Medical University of Graz, Austria. Two hundred subjects with 25(OH)D concentrations < 30 ng/mL and arterial hypertension were randomized to receive either 2800 IU of vitamin D3 daily or placebo over 8 weeks. Primary outcome was the between-group difference in FGF23 levels at study end while adjusting for baseline values.

**Results:**

Overall, 181 participants (mean ± standard deviation age, 60.1 ± 11.3; 48% women) with available c-term FGF23 concentrations were considered for the present analysis. Mean treatment duration was 54 ± 10 days in the vitamin D3 group and 54 ± 9 days in the placebo group. At baseline, FGF23 was significantly correlated with serum phosphate (*r* = 0.135; *p* = 0.002). Vitamin D3 supplementation had no significant effect on FGF23 in the entire cohort (mean treatment effect 0.374 pmol/L; 95% confidence interval − 0.024 to 0.772 pmol/L; *p* = 0.065), but increased FGF23 concentrations in subgroups with baseline 25(OH)D concentrations below 20 ng/mL (*n* = 70; mean treatment effect 0.973 pmol/L; 95% confidence interval − 0.032 to 1.979 pmol/L; *p* = 0.019) and 16 ng/mL (*n* = 40; mean treatment effect 0.593 pmol/L; 95% confidence interval 0.076 to 1.109; *p* = 0.022).

**Conclusions:**

Vitamin D3 supplementation had no significant effect on FGF23 in the entire study cohort. We did, however, observe an increase of FGF23 concentrations in subgroups with low baseline 25(OH)D.

## Introduction

Fibroblast growth factor-23 (FGF23) is a major regulator of calcium and phosphate homeostasis. Together with calcitriol [1,25(OH)_2_D3] and parathyroid hormone (PTH), it is part of a complex multi-tissue feedback system [1]. By reducing the expression of sodium-phosphate cotransporters (Npt2a and Npt2c), FGF23, in conjunction with its cofactor klotho, exerts phosphaturic effects on the kidney. It also impairs formation of calcitriol and decreases PTH mRNA in the parathyroid glands [2]. In contrast, increasing levels of calcitriol stimulate the secretion of FGF23 [3]. While this cause and effect relationship is not yet established, elevated serum concentrations of FGF23 are associated with increased mortality in hemodialysis patients [4] and patients with stable coronary artery disease [5, 6] and are furthermore related to progression of left ventricular hypertrophy and renal disease as well as increased fat mass and dyslipidemia in elderly patients [7, 8].

Since vitamin D supplementation leads to a suppression of PTH and an increase in calcitriol concentrations [9, 10], a resulting rise in serum phosphate levels may in turn stimulate the synthesis of FGF23. Considering its potential adverse effects on various health outcomes, several previous studies aimed to investigate a possible effect of vitamin D supplementation on FGF23 concentrations. However, these studies yielded inconsistent results and were based on relatively small sample sizes. In a study by Burnett-Bowie et al. [11] in 90 18- to 45-year-old subjects, treatment with 50,000 IU of vitamin D2 (ergocalciferol) weekly in subjects with 25-hydroxyvitamin D [25(OH)D] levels ≤ 20 ng/mL (multiply by 2.496 to convert ng/mL to nmol/L) increased FGF23 when compared to the placebo group. In another study conducted in 40 healthy participants with 25(OH)D levels ≤ 20 ng/mL [12], there was a significant within group increase in FGF23 in the intervention group receiving 3000 IU of vitamin D3 (cholecalciferol) daily over 4 months, but no significant difference in changes in FGF23 between the intervention and the placebo group. In overweight/obese African–American subjects with a 25(OH)D level ≤ 20 ng/mL (*n* = 70), no dose- or time-dependent changes in either FGF23 or phosphorus after supplementation with either 18,000, 60,000, or 120,000 IU of vitamin D3 monthly over 16 weeks could be observed [13].

In the present study we aimed to evaluate the effect of vitamin D3 supplementation on FGF23 concentrations by performing a post-hoc analysis of a vitamin D randomized-controlled trial (RCT) in 200 hypertensive patients with 25(OH)D levels < 30 ng/mL. Our main study aim was to evaluate whether vitamin D3 supplementation as compared to placebo has an effect on FGF23 levels in the entire study population, as well as in subgroups with particularly low 25(OH)D levels.

## Methods

### Design

The present study is a post-hoc analysis of the Styrian Vitamin D Hypertension trial, a single-center, double-blind, placebo-controlled, parallel-group study that was conducted at the Medical University of Graz, Austria, from June 2011 until August 2014 [14]. Methods and design of this trial have been published previously [14]. The clinical trial is registered on http://www.clinicaltrialsregister.eu (EudraCT number 2009-018125-70) and on clinicaltrials.gov (ClinicalTrials.gov Identifier NCT02136771). Publication of data from this trial adheres to the Consolidated Standards of Reporting Trials (CONSORT) 2010 statement [15].

### Participants

To be eligible for study participation, subjects needed to be 18 years of age or older with diagnosed arterial hypertension and a serum concentration of 25(OH)D below 30 ng/mL. Arterial hypertension was defined in adherence to existing guidelines [16] or by ongoing intake of antihypertensive therapy. Exclusion criteria for the trial were regular intake of > 880 IU of vitamin D3 daily within 4 weeks before inclusion in the trial (assessed by patient interview or previous medical reports), 24-h systolic blood pressure > 160 mmHg or < 120 mmHg, 24-h diastolic blood pressure > 100 mmHg, change of antihypertensive therapy within the previous 4 weeks or planned changes of antihypertensive therapy, hypercalcemia defined as a plasma calcium concentration > 2.65 mmol/L, pregnant or lactating women, drug intake as part of another clinical study, acute coronary syndrome, cerebrovascular events within the previous 2 weeks, an estimated glomerular filtration rate according to the Modification of Diet in Renal Disease formula < 15 mL/min per 1.73 m^2^ [17], any clinically significant acute disease requiring drug treatment, chemotherapy or radiation therapy, or any disease with an estimated life expectancy of less than 1 year.

### Intervention

The study medication was placed into numbered bottles adhering to a computer-generated randomization list provided by a web-based software (http://www.randomizer.at) with compliance to good clinical practice as confirmed by the Austrian Agency for Health and Food Safety. Participants were randomly allocated in a 1:1 ratio to either receive 2800 IU of vitamin D3 (Oleovit D3, Fresenius Kabi Austria, Austria) or a matching placebo (each as seven oily drops per day for a total duration of 8 weeks). We performed permuted block randomization with a block size of 10 and stratification according to sex. All investigators/authors who enrolled participants, collected data, and assigned intervention were masked to participant allocation.

### Outcome measure

The current study is a post-hoc analysis that investigates the between-group differences in FGF23 levels at study end while adjusting for baseline values [18].

### Measurements

Physical examination, blood sampling, and patient interviews were conducted at both study visits between 7 and 11 AM after an overnight fast of at least 12 h and a sitting resting period of at least 10 min, without smoking on the day of blood sampling. The study participants then left the hospital for ambulatory blood pressure measurements and 24-h urine collections before they returned to the outpatient department on the following day. At this point, eligible participants were randomized and started with the intake of the study medication. At both study visits, 22 ml of sampled blood were frozen and stored at − 80 °C at the laboratory of our department for future laboratory measurements. For the present post-hoc analysis, FGF23 was measured from these samples between January 13th and January 20th 2016. FGF23 was measured by a multi-matrix ELISA (FGF23 (C-terminal) ELISA; BIOMEDICA Medizinprodukte GmbH & CO KG, Vienna, Austria) with an intra-assay and inter-assay coefficient of variation (CV) of ≤ 12 and ≤ 10%, respectively. For the current analysis, FGF23 was measured from plates with mixed samples (i.e., no separation in regard to study group or study visit). For our measurements, we calculated intra- and inter-assay CVs of 5.6 and 7.4%, respectively. 25(OH)D was measured by a chemiluminescence assay (IDS-iSYS 25-hydroxyvitamin D assay; Immunodiagnostic Systems Ltd, Boldon, United Kingdom) with an intra-assay and inter-assay CV of 6.2 and 11.6%, respectively. Details on several other laboratory measurements haven been published previously [14, 19, 20].

### Data analysis

Continuous data following a normal distribution are shown as means with standard deviations (SDs), while parameters with a skewed distribution are shown as medians with interquartile ranges. Categorical data are presented as percentages. Where appropriate, skewed variables were log(e) transformed before they were used in parametric analyses. Comparisons between the vitamin D and placebo group at baseline were calculated by the unpaired Student’s *t* test, the Mann–Whitney-*U* test, or Chi-square test, whichever was appropriate. Pearson correlation analysis was performed to evaluate the correlation of baseline levels of FGF23 and phosphate. Analysis of covariance with adjustments for baseline values of FGF23 was used to test for differences in FGF23 levels between the treatment and the placebo group at follow-up visit [18]. Analysis of covariance was also performed in subgroups with baseline 25(OH)D levels below 20 and 16 ng/mL, as these values are regarded to cover the requirements of 97.5 and 50% of the population, respectively [21]. Analyses were performed according to the intention-to-treat principle with no data imputation and inclusion of all participants with baseline and follow-up values of the respective outcome variable. A *p* value < 0.05 was considered statistically significant. All statistical operations were performed with SPSS version 23 (SPSS, Chicago, IL, USA).

## Results

About 1700 persons were invited to participate in the study, of which 518 gave written informed consent and were, therefore, assessed for eligibility. Randomization as well as follow-up visits took place between June 2011 and August 2014. For this post-hoc analysis of the trial, only participants with available values of FGF23 at both the baseline and follow-up visit were considered [*n* = 181; mean (SD) age, 60.1 (11.3) years; 48% women; baseline 25(OH)D, 21.3 (5.6) ng/mL].

Baseline characteristics of the included participants are shown in Table 1. At baseline, serum levels of FGF23 were significantly higher in the placebo group when compared to the treatment group. All other baseline parameters showed no significant differences between the groups (Table 1). The mean treatment period was 54 ± 10 days in the vitamin D3 group and 54 ± 9 days in the placebo group.

**Table 1:** Baseline characteristics of all randomized participants with available FGF23 values at both baseline and follow-up visit

	All (*n* = 181)	Vitamin D (*n* = 90)	Placebo (*n* = 91)	*p* value
Age (years)	60.1 ± 11.3	60.6 ± 10.9	59.5 ± 11.6	0.492
Females (%)	48	46	51	0.553
BMI (kg/m^2^)	30.2 ± 5.1	30.4 ± 4.4	30.1 ± 5.7	0.709
Office systolic BP (mmHg)	142.3 ± 15.1	142.2 ± 14.6	142.4 ± 15.6	0.948
Office diastolic BP (mmHg)	86.6 ± 10.3	86.5 ± 9.9	86.7 ± 10.7	0.881
FGF23 (pmol/L)	0.795 (0.582–1.211)	0.723 (0.542–1.118)	0.894 (0.636–1.286)	0.024
25(OH)D (ng/mL)	21.3 ± 5.6	22.1 ± 5.4	20.5 ± 5.8	0.053
Calcitriol (pg/mL)	49.9 ± 18.8	52.0 ± 18.4	47.9 ± 19.0	0.138
PTH (pg/mL)	49.0 (39.6–62.5)	48.9 (39.6–61.4)	51.3 (39.3–63.5)	0.884
Serum calcium (mmol/L)	2.37 ± 0.10	2.37 ± 0.10	2.37 ± 0.11	0.898
Serum phosphate (mmol/L)	0.95 ± 0.16	0.93 ± 0.17	0.97 ± 0.16	0.167
Creatinine (mg/dL)	0.87 (0.76-1.00)	0.87 (0.75–0.98)	0.88 (0.76–1.03)	0.394
eGFR (mL/min/1.73 m)	79.0 ± 18.0	80.6 ± 18.1	77.6 ± 17.8	0.261
Triglycerides (mg/dL)	121 (82–168)	123 (82–168)	119 (81–163)	0.741
HDL-cholesterol (mg/dL)	56.5 ± 16.5	55.7 ± 16.5	57.2 ± 16.5	0.518
LDL-cholesterol (mg/dL)	114.0 ± 40.0	115.8 ± 41.1	112.3 ± 39.0	0.564
CRP (mg/L)	1.8 (0.9–3.5)	2.1 (0.9–3.8)	1.4 (0.8-3.0)	0.086
Use of additional vitamin D supplements (%)^a^	7.7	5.6	9.9	0.275

Data are shown as means with standard deviation, median and interquartile range, or as percentages, whichever was appropriate. Comparisons between the vitamin D and placebo group were calculated by Student *t* test, Mann–Whitney-*U* test, or Chi-square test

*BMI* body-mass index, *BP* blood pressure, *FGF23* fibroblast growth factor 23, *25(OH)D* 25-hydroxyvitamin D, *PTH* parathyroid hormone, *eGFR* estimated glomerular filtration rate, *HDL*-cholesterol high-density lipoprotein-cholesterol, *LDL*-cholesterol low-density lipoprotein-cholesterol, *CRP* C-reactive protein

^a^< 880 IU of cholecalciferol daily

At baseline, FGF23 was significantly correlated with serum phosphate (*r* = 0.135; *p* = 0.002). In the vitamin D3 group, 25(OH)D significantly increased with a mean treatment effect [95% confidence interval (CI)] of 11.1 (8.9–13.3) ng/mL (*p* < 0.001). Vitamin D3 supplementation had no significant effect on FGF23 concentrations after adjustment for baseline values of FGF23 [mean treatment effect (95% CI), 0.374 (− 0.024 to 0.772) pmol/L; *p* = 0.065]. However, in subgroup analysis, vitamin D3 supplementation lead to a significant increase in FGF23 levels in subgroups with 25(OH)D levels below 20 ng/mL (*n* = 70) and 16 ng/mL (*n* = 40) at baseline (Table 2).

**Table 2:** FGF23 at baseline and follow-up in all participants and subgroups with low baseline 25(OH)D concentrations

	Baseline	Follow-up	Treatment effect	*p* value
FGF23 (pmol/L), all participants (*n* = 181)				
Vitamin D (*n* = 90)	0.723 (0.542–1.118)	0.824 (0.598–1.168)	0.374 (− 0.024 to 0.772)	0.065
Placebo (*n* = 91)	0.894 (0.636–1.286)	0.944 (0.641–1.231)		
FGF23 (pmol/L), 25(OH)D < 20 ng/mL (*n* = 70)				
Vitamin D (*n* = 30)	0.708 (0.519–1.051)	0.780 (0.562–1.385)	0.973 (− 0.032 to 1.979)	0.019
Placebo (*n* = 40)	0.987 (0.644–1.528)	0.927 (0.651–1.260)		
FGF23 (pmol/L), 25(OH)D < 16 ng/mL (*n* = 40)				
Vitamin D (*n* = 15)	0.718 (0.494–1.020)	0.743 (0.570–1.601)	0.593 (0.076 to 1.109)	0.022
Placebo (*n* = 25)	0.971 (0.694–1.360)	0.879 (0.635–1.201)		

Data are shown as medians and interquartile ranges. Treatment effects with 95% confidence interval and *p* values were calculated by ANCOVA for group differences at follow-up with adjustment for baseline values. FGF23 was logarithmic transformed before values were used in ANCOVA, but untransformed values are shown in the table

*FGF23* fibroblast growth factor 23, *25(OH)D* 25-hydroxyvitamin D

There was no excess of adverse events (i.e., hypercalcemia or hospitalization) in the vitamin D3 group, no patient died during study participation. No patient in the treatment group developed hypercalcemia at the final study visit.

## Discussion

In this RCT in hypertensive patients, we did not observe a significant effect of vitamin D3 supplementation on FGF23 in our entire study cohort. In subgroups with 25(OH)D concentrations below 20 and 16 ng/mL, vitamin D3 supplementation lead to a significant increase in FGF23 concentrations.

Our findings on vitamin D effects on FGF23 significantly extend previous study data that were, however, limited by, e.g., relatively low sample sizes or statistical analyses lacking adequate tests for between-group differences. In a study by Alshayeb et al. [3], treatment with 10,000 IU of vitamin D3 weekly over 8 weeks lead to a significant increase in FGF23 concentrations in subjects without chronic kidney disease (CKD) (*n* = 25; eGFR > 60 mL/min/1.73 m^2^). Burnett-Bowie et al. [11] observed a significant increase in FGF23 in the treatment group after supplementing 50,000 IU of vitamin D2 weekly in healthy subjects (*n* = 90) over 12 weeks, while Nygaard et al. [12] reported a significant increase in FGF23 after supplementing 3000 IU of vitamin D3 daily over 4 months in 40 healthy participants. In contrast, a study conducted in African Americans with low 25(OH)D concentrations (≤ 20 ng/mL; *n* = 70) was unable to find any significant dose- or time responses on FGF23 after treatment with 18,000–120,000 IU of vitamin D3 monthly over 16 weeks [13].

The inconsistency in the literature regarding effects of vitamin D supplementation on FGF23 concentrations could be a result of different dosing regimens, e.g., monthly vs. weekly vs. daily supplementation of vitamin D or due to differences in study participant characteristics as well as sample sizes. The fact that we were able to find a significant increase in FGF23 levels in subgroups with baseline 25(OH)D concentrations below 20 and 16 ng/mL but could not find a similar effect in the entire study cohort may be explained by a possibly higher increase in calcitriol in participants with lower baseline 25(OH)D concentrations [9], thus leading to a more pronounced rise in FGF23 concentrations due to increased intestinal phosphate absorption [22]. A significant increase in calcitriol concentrations after vitamin D3 supplementation within the population of the Styrian Vitamin D Hypertension trial has been reported previously [10].

The observed effect of vitamin D3 on FGF23 in participants with 25(OH)D below 20 ng/mL may be of clinical importance, because vitamin D supplements are, in general, prescribed or indicated in individuals with low 25(OH)D concentrations. Elevated serum concentrations of FGF23 were associated with increased mortality as well as other adverse health outcomes, e.g., progression in left ventricular hypertrophy and renal disease [4–8]. Whether the increase in FGF23 in our subgroup analyses can be considered a beneficial defense mechanism against vitamin D induced phosphate increases or whether this increase in FGF23 may even be harmful is speculatively and warrants further in depth investigations, especially in view of the rising relevance of FGF23 as a biomarker [23].

At baseline, we observed a significant correlation of FGF23 with serum phosphate. However, the mechanisms of the regulation of FGF23 levels by serum phosphate are currently not entirely understood. This is underscored by the fact that even though phosphate levels are positively correlated with elevations in FGF23 in CKD patients, phosphate restriction did not lower FGF23 concentrations in these patients [24, 25]. Data in patients without CKD are currently conflicting, as some studies report weak correlations of serum phosphate with FGF23 [26], while others were unable to find significant results [27]. The weak, yet significant, correlation of serum phosphate with FGF23 in our study population may also be explained by the relatively narrow range and lower levels of serum phosphate in our study cohort as compared to CKD populations.

A possible limitation of our study is its restriction to patients with arterial hypertension and 25(OH)D concentrations < 30 ng/mL. Its results may be, therefore, not uncritically generalizable to the general public or specific patient groups of interest, e.g., patients on hemodialysis. Another limitation is the use of an immunoassay to measure 25(OH)D concentrations instead of standardized measurements such as in the Vitamin D Standardization Program (VDSP). In addition, only 70 and 40 participants showed baseline 25(OH)D levels below 20 and 16 ng/mL respectively, therefore, significantly limiting the sample size of our subgroup analyses. More so, subgroup and post-hoc analyses of RCTs have intrinsic weaknesses and the derived findings must be interpreted with caution [28]. The strengths of this study are its design as a RCT and its well-documented treatment effects on 25(OH)D and PTH [14]. Due to its design, our results significantly add to the preexisting data in the field. Moreover, the validity of our data is underscored by the confirmation of the well-known association between FGF23 and serum phosphate [29].

In conclusion, we did not observe a significant effect of vitamin D3 supplementation on FGF23 concentrations in patients with vitamin D insufficiency and arterial hypertension. There was, however, a significant rise in FGF23 in subgroups with baseline 25(OH)D concentrations below 16 and 20 ng/mL, respectively. Further studies in the general population and in patient groups of particular interest (e.g., hemodialysis patients) are still warranted to gain further insight on the interaction of vitamin D supplementation and FGF23 as well as its clinical implications.
